# Design and Test of a Microdestructive Tree-Ring Measurement System

**DOI:** 10.3390/s20113253

**Published:** 2020-06-07

**Authors:** Xueyang Hu, Yili Zheng, Hao Liang, Yandong Zhao

**Affiliations:** 1 School of Technology, Beijing Forestry University, Beijing 100083, China; huxueyang@bjfu.edu.cn (X.H.); lianghao@bjfu.edu.cn (H.L.); yandongzh@bjfu.edu.cn (Y.Z.); 2 Beijing Laboratory of Urban and Rural Ecological Environment, Beijing 100083, China; 3 Key Lab of State Forestry Administration for Forestry Equipment and Automation, Beijing 10083, China

**Keywords:** analysis of a tree ring, DC servomotor control, FIR filtering, microdestructive measurement

## Abstract

Analysis of a tree ring is the primary method for determining the growth and age of a tree. In a microdestructive tree-ring measurement system, the tree under test is drilled with a microdrill at a constant rotating speed to detect the difference in density between the early and late wood, thereby realizing a microdestructive measurement of the tree-ring. The measurement system comprises a microdrill with a diameter of 3 mm, mechanical transmission, direct current (DC) servomotor, stepper motor, and control and detection circuit. The DC servomotor and stepper motor realize rotation and translation of the microdrill, respectively, through mechanical transmission. When the microdrill rotates and drills into the tree, the control and detection circuit samples and acquires the armature current of the DC servomotor, which is proportional to the resistance encountered by the drill bit and reflects the change in the density of the tree. The tree-ring number can be obtained by filtering the sampled original signals of the armature current using a finite impulse response (FIR) filtering algorithm. The annual rings of larch and fir tree discs were measured and tested using the designed system. It was observed that the average annual ring measurement accuracy of the larch discs reached 95.28%, while that of the fir discs was 84.16%. The diameter of the drill hole in the trunk was less than 3 mm after measuring the living wood, thereby achieving a microdestructive measurement of the tree-ring.

## 1. Introduction

Trees have different periodic seasonal growth speeds and form annual rings with a concentric ring-shaped structure on the xylem cross-section. The annual rings consist of early and late wood, which have significantly different densities. Through the analysis of tree rings, the growth and health of the living wood can be monitored. Moreover, the inherent information contained in the annual rings can be utilized to recover or reconstruct the evolution process of the natural environment. Additionally, the age of live trees, logs, and wood products as well as old and valuable trees and ancient buildings can be determined accurately [1,2]. Dendrochronology has developed into an interdisciplinary discipline that is widely used in the fields of forestry, ecology, climatology, and environmental science [3,4].

At present, the tree ring measurement techniques can be divided into three types, i.e., destructive, nondestructive, and microdestructive detection. The destructive annual ring detection employs the traditional disc cutting and wood core drilling methods, using a growth cone for sampling and analysis [5]. The main problem with this type of method is that it causes significant damage to trees. The primary disadvantage of the disc cutting method is the substantial damage to the tree from cutting the tree trunk discs, which makes it the difficult for the tree to survive. Thus, this method is inapplicable for measuring the annual rings of valuable old trees and rare tree species. Moreover, the transportation is inconvenient in most forest regions, which results in relatively high transportation and preservation costs of the disc samples. The wood core drilling method uses a special tree growth cone to drill out the wood core of the tree under test, but the damage to the tree is still relatively significant [6]. Taking the growth cone developed by Haglöf Sweden as an example, the inner diameter of the damage caused by wood core drilling is approximately 10 to 12 mm, which is large enough to damage the sieve tubes that transport nutrients in the tree, thereby increasing the risk of disease in the tree and affecting its survival rate [7].

The nondestructive annual ring detection methods mainly include computed tomography (CT) techniques and stress wave detection technology. The main problem with this type of method is that the recognizability is low. Cerda et al. proposed an automatic annual ring identification and detection method, the computed tomography technique based on computer vision, which can reconstruct the cross-sectional image of a tree without causing any damage [8,9]. However, due to the complex internal structure of a tree and the varying annual ring patterns, the CT images have an uneven grayscale, and circular artifacts are likely to occur, thereby reducing the recognizability of the annual rings. At the same time, the CT instrument is cumbersome and exposes the operators to radiation hazards, making it inconvenient to for real-time measurements in outdoor forest regions [10,11]. Ross et al. adopted the stress wave detection technique to study the elastic modulus of wood, and the relationship between stress wave speed and wood density. The detection of the tree density can be realized using this stress wave technique. However, this detection equipment has relatively low detection accuracy, and it can only identify the rot and holes present in the tree, but not the annual rings or other small density changes [12,13].

The needle measuring approach is often adopted in the microdestructive annual ring detection methods, in which a hole is drilled in the tree using a microdrill. The resistance parameter of the drill needle in the wood is collected through a microcomputer system, and an image of the resistance curve is displayed [14]. Rinn et al. used the needle resistance device to detect the wood from different tree species in a dry state and determine its decay condition. Their results showed that the decay location could be determined preliminarily according to the differences in the resistance curve, apparent decreasing trend, and curve fluctuation amplitude. In addition, the decay level of the tested wood can be established, and, with the knowledge of wood science, the early/late wood density and annual ring number can be determined [15].

In 2011, Kraler et al. measured trees and timber constructions using the IML-RESI F400S Resistograph [16]. The experimental results show that, under bad conditions, even the 1.5 mm annual ring width cannot be identified. In 2015 and 2016, Szewczyk et al. used the IML-E400 Resistograph, a type of needle measuring equipment, to test the annual rings of 15 pine trees, including larch and conifer. They found that the age of pine trees measured using the IML-E400 Resistograph was an average of 6.5 years younger than the age of trees measured by the growth cone method [17]. These experiments show that the accuracy of the current equipment based on the needle measuring method has not achieved the expected effect.

To resolve the problems of tree damage, low accuracy, and poor portability of the existing destructive and nondestructive annual ring detection methods, and further improve the detection accuracy of the existing needle measuring equipment, this study designed a new type of system, the microdestructive tree-ring measurement system, based on the needle measuring method and verified its performance by testing.

The contributions of this study are summarized as follows:

1. A complete design scheme of the dual motor discrete transmission structure and the digital signal processor (DSP) control and detection circuit of the microdestructive tree-ring measurement system is proposed.
2. The finite impulse response (FIR) filtering algorithm used in the field of communication and image processing was innovatively applied to the signal processing process of the microdestructive tree-ring measurement system and achieved good results.
3. The microdestructive tree ring detection is realized by this system, and its maximum damage diameter is only 3 mm.
4. The high accuracy and efficiency of the system are verified by testing larch and fir discs.

The remainder of this paper is organized as follows. Section 2 explains the microdestructive tree-ring measurement system. Section 3 describes the mechanical structure design, especially the transmission structure and the selection of motor. Section 4 presents the design of the control and detection circuit based on the DSP main control chip. Section 5 shows the signal processing method using the FIR filtering algorithm. Section 6 presents and analyzes the results of the test for system accuracy. Finally, the outcome of this work is summarized in Section 7.

## 2. Working Principle

**Figure 1:**
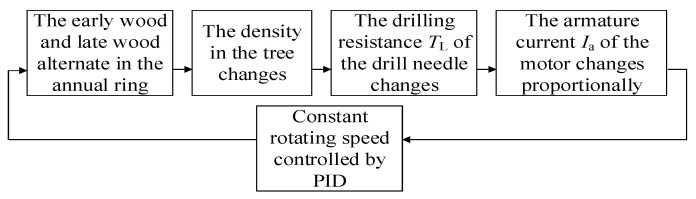
The working principle diagram.

Due to the difficulty in measuring the resistance of the microdrill directly, we chose to use the motor armature current as the indirect measurement of resistance in our system. The working principle of the microdestructive tree-ring measurement system is shown in Figure 1. The system utilizes a direct current (DC) servomotor to drive the microdrill to rotate and drill into the tree and a proportion integration differentiation (PID) servo controller to make the drill needle rotate at a constant speed [18]. In the uniform-speed drilling process, the resistance torque $T_{L}$ encountered by the microdrill bit will change when it comes into contact with the tree’s early wood and late wood, which have different densities.

Equations (1) and (2) are the torque equilibrium equation and torque characteristics of the DC brushed motor.

(1) $$T=T_{L}+T_{0}+J\frac{d\Omega }{dt}$$

(2) $$T=C_{t}\phi I_{a}$$

T is the electromagnetic torque. $T_{L}$ is the load torque. $T_{0}$ is the no-load torque. J is the moment of inertia. $J\frac{d\Omega }{dt}$ is the inertia torque. $C_{t}$ is the torque constant. ϕ is the flux per pole, which is determined by the characteristics of the motor. $I_{a}$ is the armature current.

Equation (3) can be obtained from Equations (1) and (2).

(3) $$T_{L}+T_{0}+J\frac{d\Omega }{dt}=C_{t}\phi I_{a}$$

When the system operates normally, the motor spins at a constant rotating speed; thus, the inertia torque term in Equation (3) is zero. The no-load torque $T_{0}$ is much less than $T_{L}$; therefore, the no-load torque $T_{0}$ can be neglected, and Equation (4) can then be obtained.

(4) $$T_{L}\approx C_{t}\phi I_{a}$$

In Equation (4), the armature current $I_{a}$ and resistance torque $T_{L}$ are linearly proportional. By acquiring and analyzing the changes of armature current $I_{a}$ and recording the alternating changes when the drill needle rotates and drills into the tree, the change of the resistance of the microdrill can be accurately reflected, realizing the accurate measurement of the tree-ring.

## 3. Mechanical Structure

**Figure 2:**
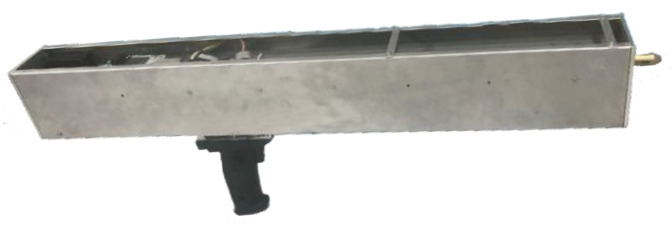
The microdestructive tree-ring measurement system.

The proposed microdestructive tree-ring measurement system is shown in Figure 2. The equipment uses a stainless-steel outer casing with a length of 87 cm, height of 29 cm, width of 9 cm, and weight of 5.3 kg. Thus, it has good portability and satisfies the requirements of outdoor forestry work.

**Figure 3:**
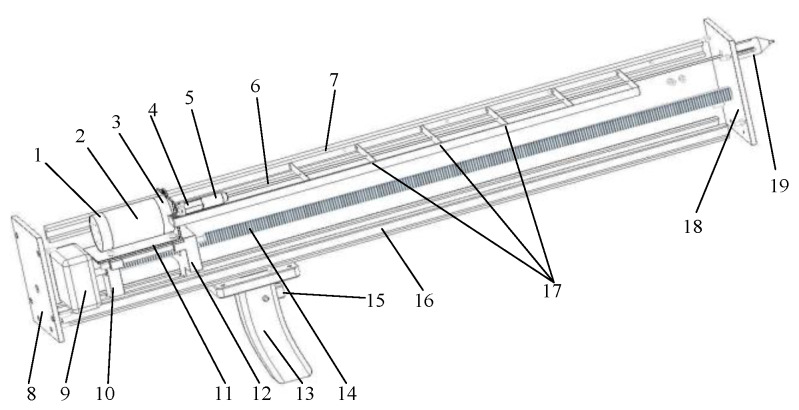
The internal structure of the microdestructive tree-ring measurement system. (1) Photoelectric rotary encoder; (2) direct current (DC) servomotor; (3) planetary gear reducer; (4) coupling; (5) drill bit clamp; (6) microdrill; (7) support plate guide; (8) back cover; (9) stepper motor; (10) rear sliding base; (11) motor base; (12) front sliding base; (13) control handle; (14) turn-screw; (15) switch button; (16) main rail; (17) support plate; (18) front cover; (19) needle copper sleeve.

The internal structure of the system is shown in Figure 3.

**Figure 4:**
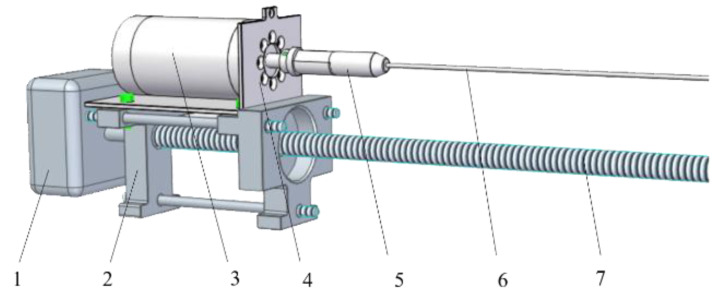
The dual motor discrete transmission structure of microdestructive tree-ring measurement system. (1) Stepper motor; (2) rear sliding base; (3) DC servomotor; (4) motor base; (5) drill bit clamp; (6) microdrill; (7) turn-screw.

In order to reduce the fluctuation of the microdrill speed, we designed a dual motor discrete transmission structure. The dual motor discrete transmission structure of the system is shown in Figure 4. The DC servomotor drives the microdrill to rotate, and the stepper motor drives the drill to move forward or backward. In the discrete dual motor drive system, the rotation speed of the microdrill is independently controlled by the DC servomotor, which makes the control effect of constant speed better. The DC servomotor is the RE35 type from Maxon Group, Switzerland; it has a rated voltage of 24 V, a maximum rotating speed of 12,000 rpm, a rated current of 3.62 A, a rated torque of 0.101 N·m, and a maximum stalled current of 41.1 A. This motor is easy to control and it has an excellent linear speed regulation. The DC servomotor is equipped with an HEDL-5540 type 1024-line incremental photoelectric rotary encoder at the tail and an all-steel planetary gear reducer at the head, with a reduction ratio of 16:1. The motor axis is connected with the microdrill through a coupling and drill bit clamp to drive the high-speed rotation of the microdrill in the tree. The DC servomotor is fixed on the sliding base through a motor base; the stepper motor connects and drives the microdrill rotation, making the sliding base move forward and backward at a precise and constant speed, thereby controlling the piercing and retrieving of the microdrill in the tree horizontally. A two-phase four-wire stepper motor is used; it has a diameter of 57 mm, a stepping angle of 1.8°, a holding torque of 1.4 N·m, a rated current of 2.8 A, and a moment of inertia of 245 g·cm^2^.

**Figure 5:**
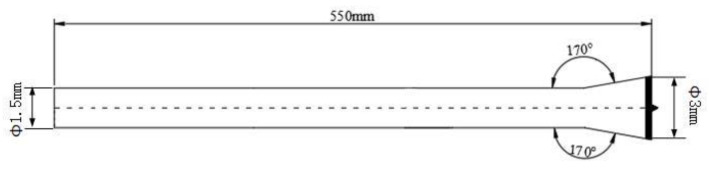
The structure of microdrill.

The microdrill adopts the structure proposed by Rinn [19], as shown in Figure 5. The maximum width of the drill bit is 3 mm, and it is designed as a flat shape with a tip. The 0.6 mm tip allows the drill bit to be drilled into the tree and identify the tree-ring density. The drill body has a diameter of 1.5 mm and is made of high-speed steel, which has a certain degree of toughness. Its total length is approximately 550 mm, which is sufficient to meet the needs for the annual ring measurement of most trees.

**Figure 6:**
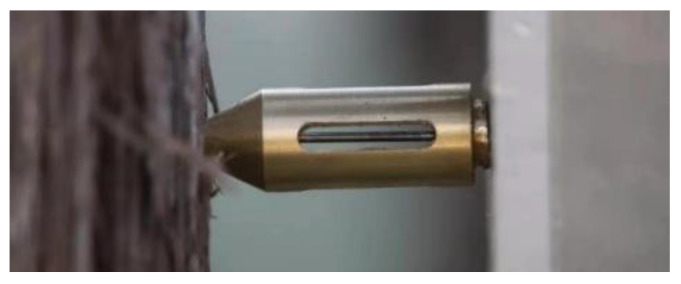
The wood-drilling experiment.

When measuring the annual rings, the DC servomotor drives the high-speed radial rotation of the microdrill at a configured rotating speed. The six support plates are evenly distributed, which can reduce the shaking and bending of the drill needle during the high-speed rotation process. The stepper motor drives the turn-screw to rotate clockwise, leading the sliding base that carries the DC servomotor to push the support board forward along the guide at a uniform speed, thereby driving the high-speed-rotating microdrill to stick out horizontally and pierce into the tree at a uniform speed to perform the measurement. When conducting the wood-drilling experiment as shown in Figure 6, the drill needle passes through the middle hole of the needle copper sleeve, and the woodchips produced during the drilling process are ejected through the side hole of the needle copper sleeve.

After the microdestructive tree-ring measurement system completes the measurement of the annual rings, the DC servomotor maintains the high-speed uniform speed rotation of the microdrill, and the stepper motor drives the turn-screw to rotate counterclockwise, leading the sliding base that carries the DC servomotor to pull back the support plate along the guide at a uniform speed. In this case, the high-speed rotating drill needle exits from inside the tree along the same path at a uniform speed, thus completing one measurement of the annual rings.

## 4. Control and Detection Circuit 

**Figure 7:**
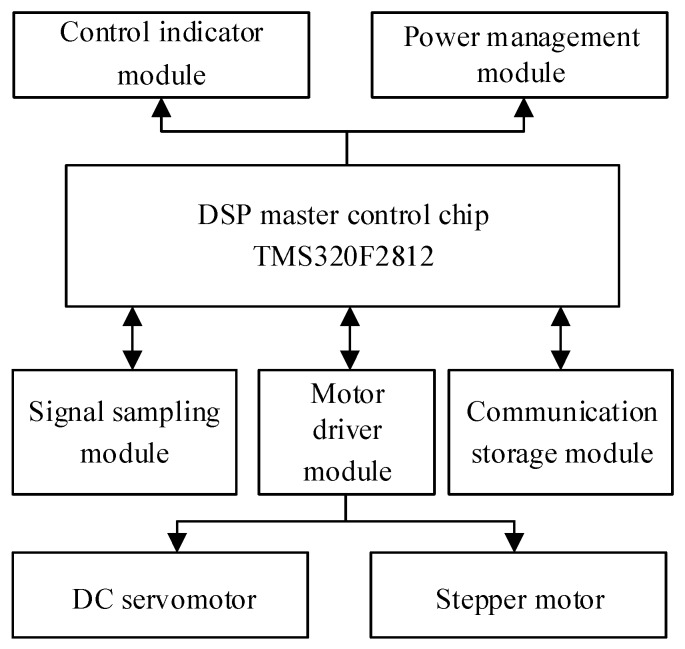
The overall structure of the control and detection circuit in the microdestructive tree-ring measurement system.

The overall structure of the control and detection circuit in the microdestructive tree-ring measurement system is shown in Figure 7, which mainly includes the DSP master control chip, motor driver, signal sampling, communication storage, control indicator, and power management modules.

The DSP master control chip module is the core of the control and detection circuit, which adopts the high-speed real-time digital signal processing system chip TMS320F2812. The built-in 12-bit high-precision analog-to-digital converter (ADC) of the TMS320F2812 achieves a high-precision sampling of the DC servomotor armature current. The 10 kHz frequency PWM adjustment output by the DSP can precisely control the motor speed. The universal asynchronous receiver/transmitter conveys the measured data. The high-performance 32-bit CPU achieves high-speed data processing [20].

The motor driver module includes the DC servomotor and stepper motor driver modules. The DC servomotor driver adopts the H-bridge driver circuit. The DSP master control chip module outputs the complementary band dead zone PWM signal to the IRS2186 driver chip. The rotary encoder provides feedback on the real-time motor rotating speed and achieves accurate speed regulation for the DC servomotor through the PID constant rotating speed control algorithm [21]. The stepper motor driver uses the TB6600 stepper motor driver, and the pulse signals sent by the DSP master control chip module control the rotating speed of the stepper motor.

The signal sampling module uses the INA282 high-precision current sampling chip, which amplifies the voltage of the precision sampling resistor that connects to the DC servomotor’s armature in series by 50 times for the high-precision sampling and analog-to-digital conversion of the TMS320F2812 ADC, which converts the armature current $I_{a}$ into the sampling voltage $U_{s}$.

The communication storage module can realize the wireless transmission of the detected sampling voltage $U_{s}$ by Bluetooth or storage in the local SD card. It uses the HC-05 Bluetooth chip, which adopts the BlueCore4 and can wirelessly transmit the sampling voltage $U_{s}$ to the host computer and display in waveform form in real time. The SD card storage module can store the sampling voltage $U_{s}$ obtained from multiple outdoor measurements in the FAT32 file format, which can be copied to a computer to perform signal processing and data analysis.

The control indicator module connects with the multiple universal I/O (input/output) ports of TMS320F2812. It can inform the operators of the working status of the equipment and send control instructions. The interactive functions include the forward and backward buttons for the microdrill, start and stop buttons for the system, buzzer alarm, and indicator lights displaying the system running status.

The power management module provides the working voltage for the system. The main power is a portable DC 24 V lithium battery pack with a capacity of 10 A·h, which can satisfy the need for the system to perform long-period outdoor operations in forest regions. The DC 24V lithium battery power supply with voltage regulator and step-down conversion circuit makes the working voltage of each functional module maintain the rated voltage when the power is reduced. This design solves the problem of the reference value of the test result changing due to power consumption, thereby improving the consistency of the test result. The B2412LS chip converts the 24 V voltage output from the lithium battery pack to a DC 12 V voltage for the IRS2186 high-end and low-end driver chip. The TPS5430 chip converts the 24 V voltage output from the lithium battery pack to a DC 7.2 V voltage, which is then converted to a DC 5 V voltage by the LM2940 for the DSP master control chip.

## 5. Signal Filtering Processing

When the microdrill is drilled into the tree, mechanical friction and vibration will be generated; in the meanwhile, the signal sampling module will inevitably introduce high-frequency noise when amplifying the signals. Thus, it is necessary to filter the raw sampling voltage $U_{s}$ signals to extract the tree-ring information accurately.

This system innovatively applies the FIR filter algorithm, widely used in the filtering and noise reduction of audio signals [22] and digital images [23], and the filtering and frequency selection in communication systems [24] for tree-ring signal processing. The FIR filtering algorithm has strict linear phase characteristics, and the pole of the system function is fixed at the origin, which results in less computational errors and more stable performance compared with infinite impulse response (IIR) filtering [25]. At the same time, computer implementation of the analytical calculation formula is easy, and the computing time is relatively short. 

**Figure 8:**
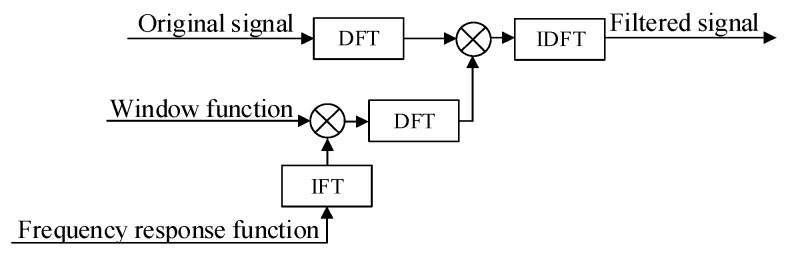
The finite impulse response (FIR) filtering algorithm flow chart.

The design methods of the FIR filtering algorithm mainly include the window function, frequency sampling, and the optimal linear phase methods [26]. The window function method has relatively simple computation, intuitive physical meaning, and widespread engineering applications. Therefore, the FIR filtering algorithm was selected for design and implementation in the current system. Figure 8 exhibits the algorithm flow chart for filtering the $U_{s}$ signal to obtain the tree-ring information.

The specific steps are as follows:

Step 1: Perform discrete Fourier transform (DFT) on the original signal. The original $U_{s}$ signal is a time-domain discrete sequence, denoted as $u_{s}(n)$. By performing DFT on $u_{s}(n)$, the corresponding frequency-domain expression $U_{s}(z)$ can be obtained, as shown in Equation (5), where n is the time-domain independent variable and z is the independent variable in the corresponding frequency domain expression.

(5) $$U_{s}(z)=DFT_{n}[u_{s}(n)]$$

Step 2: Choose the Hanning window as the filtering window function. The time-domain expression of the Hanning window function $w_{\mathrm{Hanning}}(n)$ is given in Equation (6). The Hanning window is a very useful window function. It is applicable when there is relatively strong interference noise. The tree-ring detection signal has multiple frequency components, and the spectrum performance is very complex. The purpose of the signal is to focus more on the frequency point rather than the energy. Therefore, the Hanning window is selected [27].

(6) $$w_{\mathrm{Hanning}}(n)=0.5[1-\cos(\frac{2\pi n}{N-1})]R_{N}(n)$$

N is the window length and $R_{N}(n)$ is a matrix sequence of length N.

Step 3: Construct the frequency response function. As there is a significant amount of high-frequency noise in the original signal $U_{s}$, a linear ideal low-pass filter shown in Equation (7) is selected as the frequency response function $H_{d}(e^{j\omega })$.

(7) $$H_{d}(e^{j\omega })=\{\begin{array}{c} e^{-j\omega \tau },\\ 0, \end{array}\begin{array}{c} \left|\omega \right|\leq \omega_{c}\\ \omega_{c}<\left|\omega \right|\leq \pi \end{array}$$

$\omega_{c}$ is the cut-off frequency, τ=(N−1)/2.

Step 4: Perform inverse Fourier transform (IFT) on the frequency response function $H_{d}(e^{j\omega })$ and obtain the corresponding time-domain expression $h_{d}(n)$ shown in Equation (8).

(8) $$h_{d}(n)=IFT[H_{d}(e^{j\omega })]=\frac{\sin\left[\omega_{c}(n-\tau )\right]}{\pi (n-\tau )}$$

Step 5: Apply Hanning window function $w_{\mathrm{Hanning}}(n)$ on $h_{d}(n)$ and obtain the unit impulse response h(n) of the FIR filter shown in Equation (9).

(9) $$h(n)=h_{d}(n)w_{\mathrm{Hanning}}(n)$$

Step 6: Perform DFT on the unit impulse response h(n) and obtain the FIR filter transfer function H(z) shown in Equation (10).

(10) $$H(z)={\mathrm{DFT}}_{n}[h(n)]$$

Step 7: Input $U_{s}(z)$ into H(z) and obtain the filtered signal $\bar{U_{s}(z)}$ shown in Equation (11).

(11) $$\bar{U_{s}(z)}=U_{s}(z)\cdot H(z)$$

Step 8: Perform inverse discrete Fourier transform (IDFT) on $\bar{U_{s}(z)}$ and obtain the filtered time-domain signal $\bar{u_{s}(n)}$ shown in Equation (12).

(12) $$\bar{u_{s}(n)}={\mathrm{IDFT}}_{n}[\bar{U_{s}(z)}]$$

The filtering process is completed.

**Figure 9:**
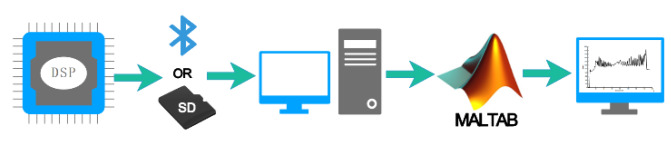
Signal processing and result display flow chart.

The FIR filtering algorithm shown above was programmed to run on the MATLAB platform. The specific steps of signal processing and result display are shown in Figure 9.

Step 1: The DSP chip encodes the sampling voltage $U_{s}$ signals and sends them to the computer through Bluetooth wireless transmission or storage of the SD card. 

Step 2: Import the received data from the computer into the MATLAB workspace.

Step 3: Run the programmed FIR filtering algorithm in the MATLAB platform to filter the data.

Step 4: The filtering result is displayed on the computer in the form of a waveform diagram.

## 6. Test

To verify that the designed system can measure the tree ring in a rapid, accurate, and microdestructive manner, larch and fir discs collected from the Jingouling Forest Farm (Yanbian Prefecture, Jilin Province, China) were used for the test.

**Figure 10:**
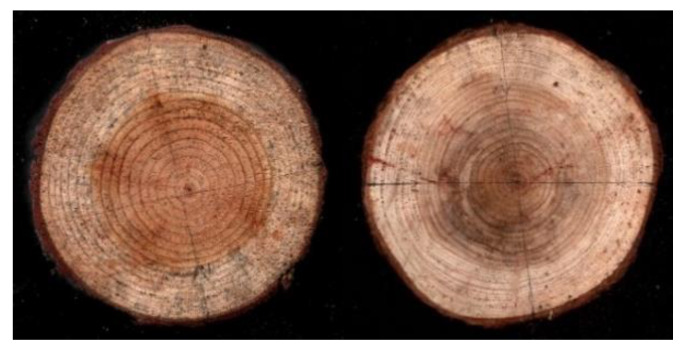
Larch #6 discs (left) and fir #5 discs (right).

Larch is a tree from the Pinaceae family. The larch discs have clear annual rings, and there is a significant difference in the densities of the early wood and late wood, making it easy to recognize the annual rings [28]. Fir is also a tree from the Pinaceae family, but the difference in density between its early wood and late wood is smaller compared to that of larch, thus requiring a higher recognition accuracy for the system. The different density of the late wood and early wood can be observed by color. A darker color of the late wood means a greater difference in density from the early wood. As shown in Figure 10, the color of larch late wood differs significantly from that of early wood. However, the late wood of fir is very narrow, and the color difference is not very obvious. The larch and fir disc samples collected from the outdoors were dried and polished for the subsequent tests.

### 6.1. Test Procedure

**Figure 11:**
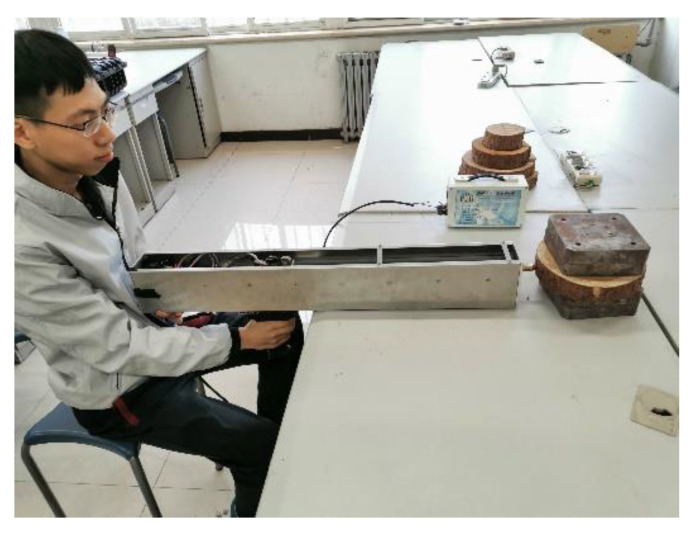
The operation of the microdestructive tree-ring measurement system.

First, the four growth directions (east, south, west, and north) on the tree discs were approximately determined according to the shape and width of the tree ring. For the same tree disc, two measurements were performed, one in the north–south direction and the other in the west–east direction, and the locations of the tree knots or disc cracks were avoided during the measurement. The pretreated discs were fixed using a vise or counterweight when performing the measurement, as shown in Figure 11.

In the test, 12 larch and 12 fir disc samples were measured, and each disc was numbered. The microdrill’s rotating speed was set at 3000 rpm, and the advancing speed was set at 10 cm/min. With the larch #6 and fir #5 discs as examples, two drilling tests were performed following the black line paths shown in Figure 10.

**Figure 12:**
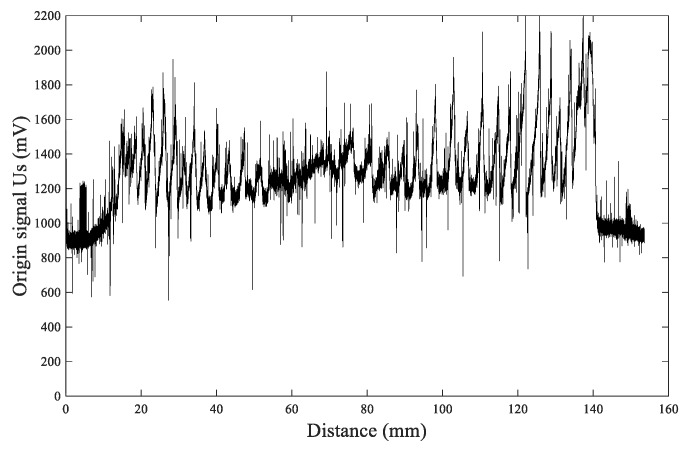
The original signal waveform of *U*_s_.

**Figure 13:**
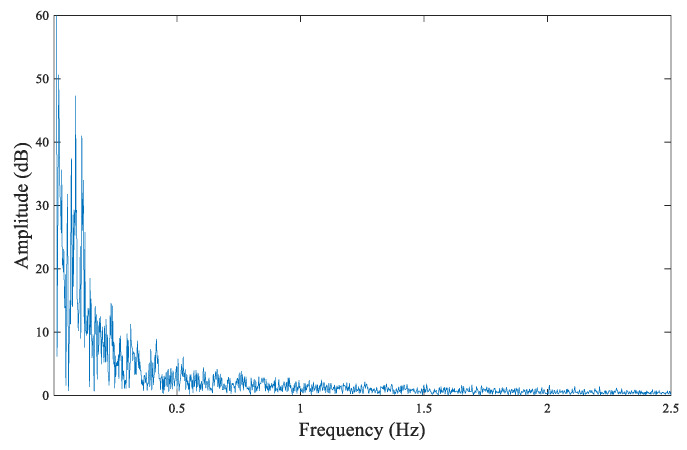
The signal frequency spectrum (0–2.5 Hz).

**Figure 14:**
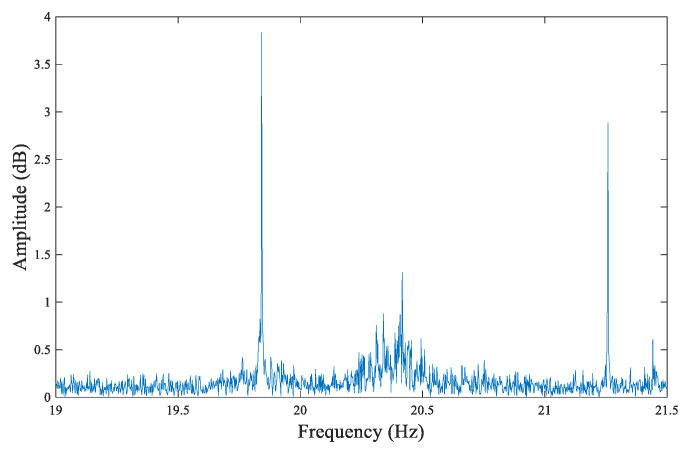
The signal frequency spectrum (19–21.5 Hz).

Taking the north–south measurement of the larch #6 disc as an example, the acquired original $U_{s}$ signal waveform plot is shown in Figure 12. By performing fast Fourier transform (FFT) on the $U_{s}$ signals, a signal frequency spectrum could be obtained, from which it can be observed that the effective signal frequencies were mainly distributed between 0 and 1.5 Hz, as shown in Figure 13; above 19.5 Hz, there was a large amount of high-frequency noise, as shown in Figure 14.

Based on the frequency spectrum of the original $U_{s}$ signal, the tree ring can be identified by filtering out the high-frequency noise through FIR low-pass filtering by MATLAB. The passband cut-off frequency was set as 1.5 Hz, as determined by the system performance and parameters and the characteristics of tree rings. The average density of tree rings was less than 6 rings per cm in our samples. When setting the advancing speed as 10 cm/min, the corresponding cut-off frequency should be 1 Hz. Considering that some tree rings are too dense in part, the cut-off frequency needs a margin above 1 Hz. However, a higher cut-off frequency introduces too much high-frequency interference, which makes it difficult for the tree rings to be identified by waveforms. Therefore, this margin cannot be too high. Setting the cut-off frequency as 1.5 Hz can meet the higher density of annual ring detection and also filter out high-frequency interference as much as possible to obtain a clear and identifiable annual ring waveform. This setting can also be verified by the analysis of Figure 13.

**Figure 15:**
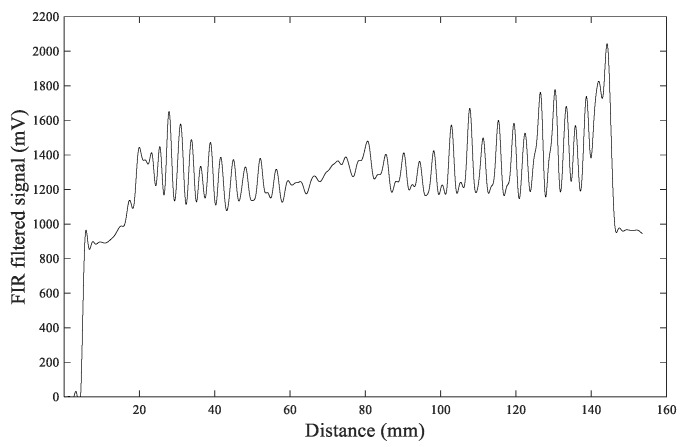
The waveform after FIR filter processing.

Figure 15 shows the waveform plot after the FIR filtering processing. The waveform shows the changes of annual rings. The annual ring number of the larch #6 disc can be calculated by counting the peaks in Figure 15.

**Figure 16:**
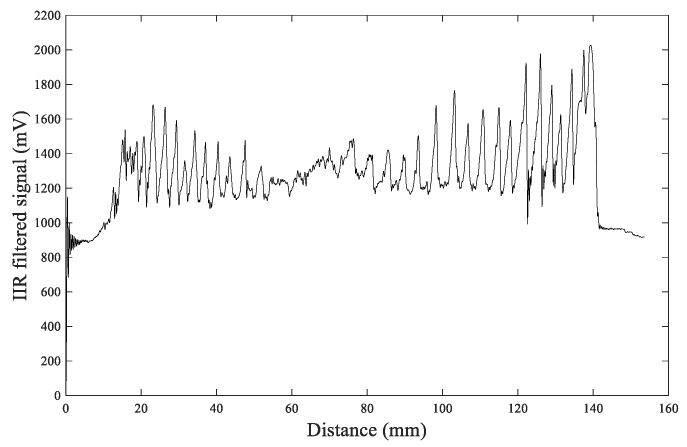
The waveform after IIR filter processing.

To verify the advantage of the FIR filtering algorithm, the IIR filtering algorithm [29] was also applied to process the original $U_{s}$ signal, and the obtained waveform after IIR filter processing is shown in Figure 16.

From the comparison of Figure 15 and Figure 16, it can be observed that the FIR filtering result is more clear and smooth and the values at the peaks and valleys are more distinct, which makes it easier to distinguish the annual rings. The IIR filtering result shows that the peaks and valleys have more dithering signals, making it challenging to identify the number of peaks, thereby resulting in errors when distinguishing annual rings. So FIR filtering is a more effective filtering algorithm.

### 6.2. Test Results

A total of 48 measurements were performed on the 12 larch and 12 fir discs. Each disc was measured in north–south and west–east directions for a total of two measurements per disc. The disc diameter, actual annual ring number, north–south and west–east annual ring measurements, and the annual ring measurement accuracy of the designed systems were recorded in the test. The results are listed in Table 1 and Table 2.

The measurement accuracy (*MA*) in the table is calculated by Equation (13).

(13) $$MA=\left(\frac{Y_{\mathrm{NS}}+Y_{\mathrm{WE}}}{2Y_{A}}\right)\times 100\%$$

$Y_{\mathrm{NS}}$ is the year number of north–south measurement. $Y_{\mathrm{WE}}$ is the year number of west–east measurement. $Y_{A}$ is the actual value.

The average measurement accuracy ($\bar{MA}$) is calculated by Equation (14).

(14) $$\bar{MA}=\frac{1}{n}\sum_{i=1}^{n}MA_{i}$$

n is the total number of tests. i is the serial number of the test. $MA_{i}$ is the measurement accuracy of the test i.

**Figure 17:**
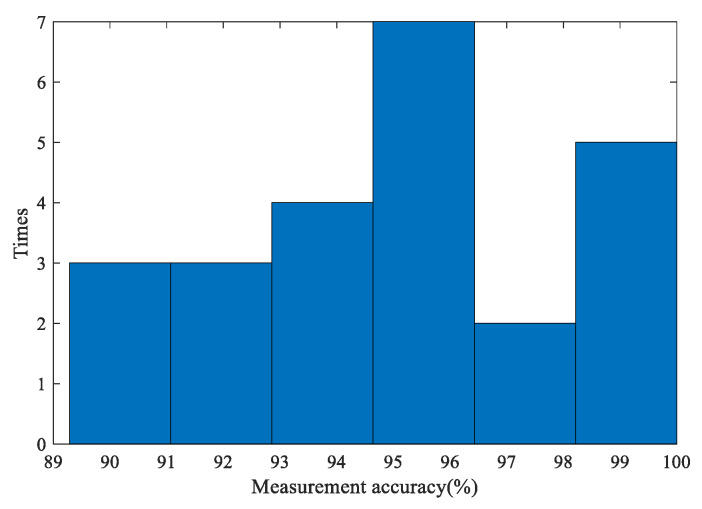
The histogram of individual larch measurement accuracies.

**Figure 18:**
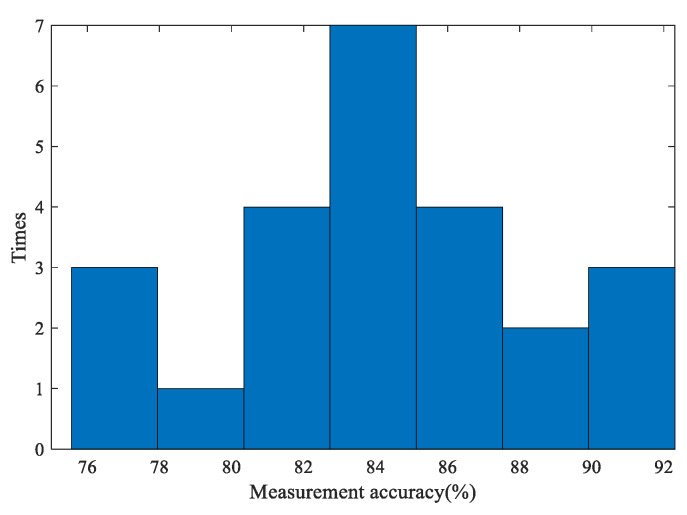
The histogram of individual fir measurement accuracies.

The individual accuracies of the 24 larch tests and 24 fir tests are presented as histograms, as shown in Figure 17 and Figure 18. In the histograms, the y-axis represents the number of measurements that correspond the range of measurement accuracy shown on the x-axis.

### 6.3. Analysis and Discussion

According to Table 1 and Figure 17, the accuracies of the annual ring measurements of the larch discs are almost higher than 90%, and the measurement accuracies (*MA*) of the larch #3 and #12 discs reached 100%. More than a quarter of the accuracies are concentrated in the 95% to 96% interval. The average measurement accuracy ($\bar{MA}$) of larch (95.28%) is also in this interval. This shows that the microdestructive tree-ring measurement system can stably obtain high accuracy. Compared with the test results of IML-E400 Resistograph average deviation of 6.5 years, the system performance for larch is very outstanding.

According to Table 2 and Figure 18, the accuracies of the annual ring measurements of the fir discs are distributed in the range of 76% to 92%. A quarter of the accuracies are concentrated in the 84% to 86% interval. The $\bar{MA}$ of fir (84.16%) is also in this interval. Obviously, the accuracy for fir is lower than larch overall.

**Figure 19:**
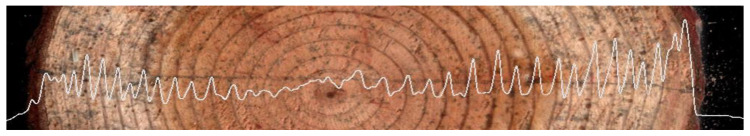
The analysis of filtered waveform and annual ring comparison of the larch #6 disk.

**Figure 20:**
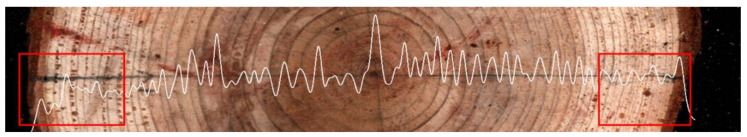
The analysis of filtered waveform and annual ring comparison of the fir #5 disk.

To verify the accuracy of the annual ring measurements and analyze the causes of the differences in the accuracy of larch and fir, the filtered waveforms measured in the north–south direction of the larch #6 and fir #5 discs were compared against the actual annual rings, as shown in Figure 19 and Figure 20.

In Figure 19 and Figure 20, the signal peaks correspond to the annual ring late wood, and the signal valleys correspond to the annual ring early wood. Every alternating occurrence of a peak and valley corresponds to an entire ring of the tree ring; thus, the tree-ring data are acquired effectively.

During the wood-drilling operation, it is challenging for the drilling path of the microdrill to pass precisely through the tree pith; therefore, there will be an error of approximately 1 to 2 center annual rings in the measurement. The same tree or disc can be further measured in different directions to reduce the error caused by this reason.

The reason for the relatively low accuracy of fir measurement is mainly due to the measurement of annual rings in the red boxes shown in Figure 20. The annual ring interval in the sapwood region of the fir disc falls approximately within the range of 0.3 to 0.5 mm. The late-wood width is less than 0.1 mm, and the annual ring distribution is relatively dense, which puts forth a higher requirement for the measurement resolution of the equipment. At the same time, when measuring the left side sapwood region of the tree, only a small portion of the microdrill can enter the tree, in which case, the relatively large interference from mechanical vibration reduced the measurement accuracy of the annual ring. These factors caused the relatively low measurement accuracy in the outer sapwood region of the fir disc annual ring. 

**Figure 21:**
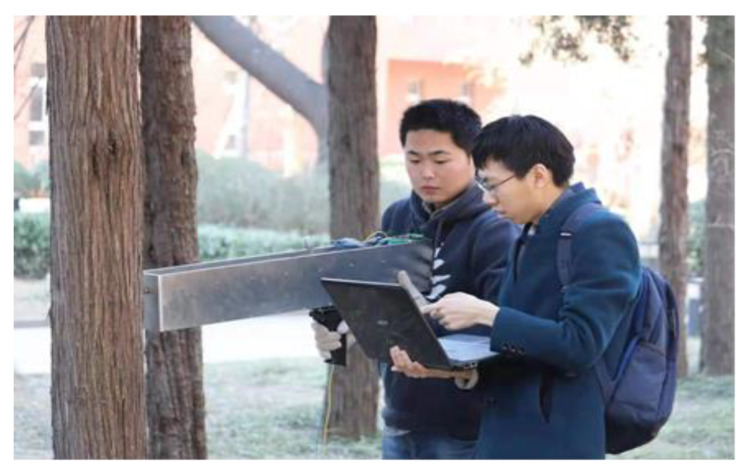
The living wood measurement.

**Figure 22:**
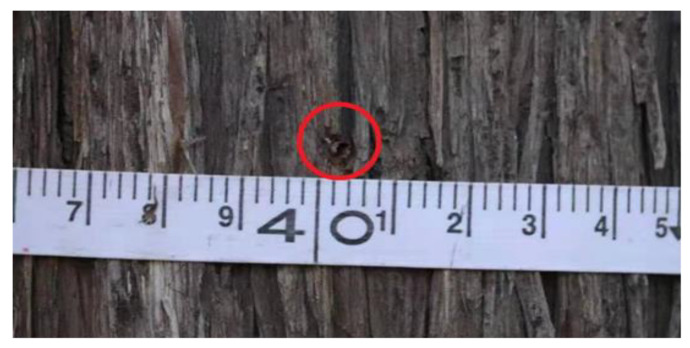
The drill hole after the measurement. The minimum length of a scale division is 2 mm.

As shown in Figure 21, the annual ring of living wood is measured using the designed system. After the measurement, the drill hole diameter in the trunk is less than 3 mm, as shown in Figure 22, which barely causes any damage to the phloem and sieve tubes that transport nutrients in the tree, thereby realizing the microdestructive measurement of the tree-ring.

Based on the analysis of the differences in the annual ring measurement accuracies for the larch and fir, we have proposed some solutions for further improving the accuracy of the system, so as to obtain better measurement results in tree species with dense annual rings and narrow width of late wood.

1. Design and machining more accurate mechanical mechanisms to reduce errors caused by mechanical vibration.
2. According to the different physical characteristics of the tree species, set different microdrill speeds and motor current sampling frequencies to make the sampling density larger.
3. According to different tree species, set different filtering parameters to more effectively filter out noise signals and retain valid signals.

## 7. Conclusions

Based on the principle that the late wood and early wood of a tree have different densities, in this study, a microdestructive tree-ring measurement system that is portable for work in forest regions was designed. The designed system uses a DC servomotor and a stepper motor to drive the microdrill to pierce into the wood and acquires the original signals of the DC servomotor armature current sampled using a DSP processor. The FIR filtering algorithm is adopted to filter the original signals and obtain the annual ring information of the tree. The average annual ring measurement accuracy of the larch discs reached 95.28%, while that of the fir discs was 84.16%. Living wood was measured, and the drill hole diameter in the trunk was less than 3 mm, thereby realizing a highly efficient and accurate microdestructive measurement of the tree rings. This system can also be applied in other fields of forestry, such as in the analysis of the decay of trees and wood density.

## Figures and Tables

**Table 1:** The measurement results for larch.

Serial Number	Tree Species	Disc Diameter (cm)	Actual Annual Ring (year)	North–South Measurement (year)	West–East Measurement (year)	Measurement Accuracy (%)
1	Larch	10.0	21	19	20	92.86
2	Larch	10.5	19	18	19	97.37
3	Larch	10.6	20	20	20	100.00
4	Larch	13.4	28	26	25	91.07
5	Larch	14.5	30	28	29	95.00
6	Larch	14.7	17	16	16	94.12
7	Larch	15.3	26	24	25	94.23
8	Larch	16.5	28	27	27	96.43
9	Larch	17.1	30	29	28	95.00
10	Larch	17.5	27	26	26	96.30
11	Larch	21.4	39	35	36	91.03
12	Larch	21.5	30	30	30	100.00

**Table 2:** The measurement results of fir.

Serial Number	Tree Species	Disc Diameter (cm)	Actual Annual Ring (year)	North–South Measurement (year)	West–East Measurement (year)	Measurement Accuracy (%)
1	Fir	11.3	21	18	17	83.33
2	Fir	13.5	27	21	24	83.33
3	Fir	14.0	24	20	21	85.42
4	Fir	14.3	28	24	23	83.83
5	Fir	15.3	31	26	24	80.65
6	Fir	15.5	26	24	22	88.46
7	Fir	16.2	31	25	26	82.26
8	Fir	16.8	45	34	36	77.78
9	Fir	17.3	33	28	29	86.36
10	Fir	17.4	22	20	19	88.64
11	Fir	18.5	36	30	29	81.94
12	Fir	23.3	33	30	28	87.88
